# Enhancement of Treatment Efficiency of Recalcitrant Wastewater Containing Textile Dyes Using a Newly Developed Iron Zeolite Socony Mobil-5 Heterogeneous Catalyst

**DOI:** 10.1371/journal.pone.0141348

**Published:** 2015-10-30

**Authors:** Mushtaq Ahmad, Anam Asghar, Abdul Aziz Abdul Raman, Wan Mohd Ashri Wan Daud

**Affiliations:** Department of Chemical Engineering, Faculty of Engineering, University of Malaya, 50603, Kuala Lumpur, Malaysia; Queen's University Belfast, UNITED KINGDOM

## Abstract

Fenton oxidation, an advanced oxidation process, is an efficient method for the treatment of recalcitrant wastewaters. Unfortunately, it utilizes H_2_O_2_ and iron-based homogeneous catalysts, which lead to the formation of high volumes of sludge and secondary pollutants. To overcome these problems, an alternate option is the usage of heterogeneous catalyst. In this study, a heterogeneous catalyst was developed to provide an alternative solution for homogeneous Fenton oxidation. Iron Zeolite Socony Mobile-5 (Fe-ZSM-5) was synthesized using a new two-step process. Next, the catalyst was characterized by scanning electron microscopy, energy-dispersive X-ray spectroscopy, fourier transform infrared spectroscopy, and Brunauer-Emmett-Teller analysis and tested against a model wastewater containing the azo dye Acid Blue 113. Results showed that the loading of iron particles reduced the surface area of the catalyst from 293.59 to 243.93 m^2^/g; meanwhile, the average particle size of the loaded material was 12.29 nm. Furthermore, efficiency of the developed catalyst was evaluated by performing heterogeneous Fenton oxidation. Taguchi method was coupled with principal component analysis in order to assess and optimize mineralization efficiency. Experimental results showed that under optimized conditions, over 99.7% degradation and 77% mineralization was obtained, with a 90% reduction in the consumption of the developed catalyst. Furthermore, the developed catalyst was stable and reusable, with less than 2% leaching observed under optimized conditions. Thus, the present study proved that newly developed catalyst has enhanced the oxidation process and reduced the chemicals consumption.

## Introduction

Wastewater discharge from the energy, mineral processing, paper, plastic, textile, and cosmetic industries contains toxic organic compounds, dyestuffs, and other recalcitrant materials which adversely affect the environment and the quality of water reservoirs [1]. The textile industry is particularly wasteful, with 125–150 L of discharged wastewater for every 1 kg of product [2]. The situation is further complicated by the fact that there are approximately 100,000 commercial dyes and pigments, available in the market and 10–15% of which find their way into the environment during the dyeing process [3]. This discharged wastewater has high levels of suspended solids and organic compounds, both toxic and otherwise, resulting in turbid, colored solutions with a wide pH range of 5–12 that in turn increase chemical and biochemical oxygen demand [4–7].

Commercial synthetic dyes are grouped into 20–30 classes based on their chemical structures or chromophores [8], with azo dyes accounting for 60–70% of these overall [9]. These dyes are characterized by one or more azo groups (-N = N-) and have poor biodegradability [10] that proceeds slowly [11]. As a result, these toxic and carcinogenic substances are always found in wastewater [12,13], necessitating intricate treatment prior to disposal.

Fenton oxidation, one of several advanced oxidation processes, is an efficient wastewater treatment method for recalcitrant wastewater. Use of iron salts and H_2_O_2_ under acidic conditions produces hydroxyl radicals (HO^●^) (Eq 1), non-selective and highly oxidative species with a redox potential of 2.80 eV, making it capable of mineralizing a wide range of recalcitrant organic contaminants [14,15]. This process is advantageous because it is characterized by high mineralization efficiency, simple operation, and short reaction times. However, the required pH and high cost of H_2_O_2_ and the excess Fenton reagents required limit the application of this process. Besides, high concentration of iron results in production of a large volume of sludge during the neutralization stage of process, which is undesirable as the concentration of iron ions in waste discharge should not exceed 2 ppm, according to limits established by the European Union [16,17].

$$Fe^{+2}+H_{2}O_{2}\to HO^{•}+Fe^{+3}+HO^{-}$$ (1)

Heterogeneous solid catalysts have been developed to overcome these issues. In the heterogeneous Fenton process, the active phase is generally immobilized on a porous support. A wide range of solid catalysts have been developed for this process, such as Fe-Cu, Al-Fe, Al-Cu, MnO_2_, Ni_2_O_3_, Pt, Fe, and Cu, which in turn can be supported on pillared clays, zeolites, or CuO, ZnO, and TiO_2_, supported on Al_2_O_3_/Al-MCM-41 have been used for heterogeneous Fenton process. However, among all these combinations, iron particles supported on a zeolite or activated carbon matrix are the most effective [18–21]. Moreover, among all the promising supports for heterogeneous process, ZSM-5 has captured the attention of many researches due to its high stability, large surface area, remarkable porosity, and unique surface chemistry. Iron particles suppress the reaction between iron and H_2_O_2_ and the produced Fe^+2^/Fe^+3^ complexes on the surface of ZSM-5 react with H_2_O_2_ to initiate the Fenton catalytic cycle [22].

Fe-ZSM-5 activity depends on the concentration, size, morphology, and surface area of the iron particles, and these parameters can normally be controlled through careful dispersion of fine iron particles through the controlled, porous structure [23]. To date, various methods have been developed and introduced to design microporous and mesoporous Fe-ZSM-5 [24], including ion exchange [25], hydrothermal processes [26], chemical vapor deposition [27], and incipient wetness impregnation [28]. Fe-ZSM-5 produced by hydrothermal process has shown better activity compared to that produced by other methods [29]; however, in all cases, metal leaching reduces catalyst activity, particularly in acidic media and at high temperature [24,26,28]. These issues must be addressed if viable catalysts for the oxidation of wastewater are to be developed [30].

The objective of this work was to study the stability and specific properties of Fe-ZSM-5 synthesized using a newly developed 2-step process with respect to heterogeneous Fenton oxidation. This method is based on a previous one used to synthesize heterometallic nanoparticles at low temperatures [31–33], and relies on the development of a metal-organic framework and its thermal decomposition in order to produce fine iron oxide particles. The efficiency of the synthesized Fe-ZSM-5 was tested against the decolorization of Acid Blue 113 and Total Organic Carbon (TOC) removal. Dye degradation trend was also evaluated at higher concentrations and in a mixture of dyes. Up to our knowledge, no one has used Fe-ZSM-5 for the degradation of Acid Blue 113.

Multiple process parameters, specifically initial dye and H_2_O_2_ concentrations, initial pH value, catalyst amount, time, and temperature, were assessed and optimized. This method improves the efficiency, stability, and reusability of the catalyst [34,35].

## Materials and Methodology

### Reagents

**Fig 1:**
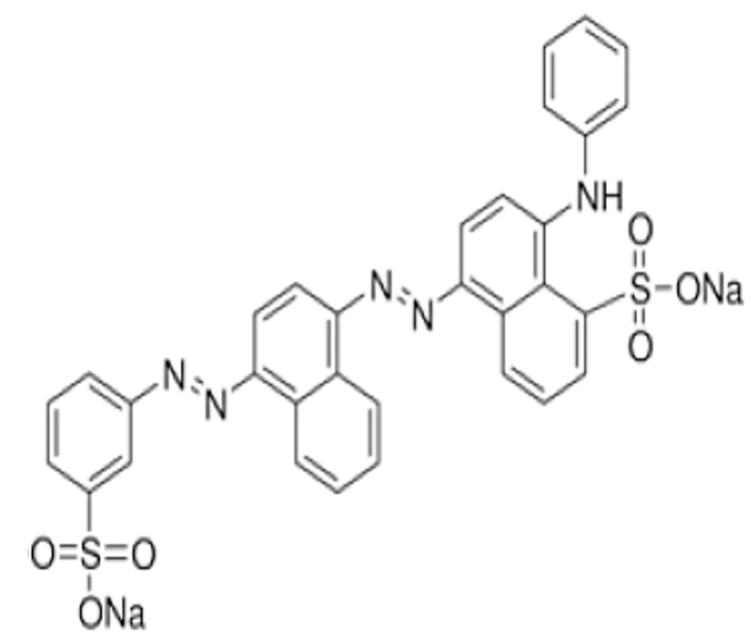
Chemical Structure of Acid Blue 113.

Acid Blue 113 was chosen as the model dye because the two azo bonds in its chemical structure, as shown in Fig 1, make it particularly recalcitrant. To make dye mixture, Methyl Orange and Reactive Black 5 were selected. All reagents were of analytical grade and used without any further purification. Dyes were procured from Sigma-Aldrich while 30% (wt/wt) H_2_O_2_ was purchased from Merck. Iron chloride, 2,2´-bipyridine, 2-propanol, and ethanol were obtained from Merck and used for the preparation of the heterogeneous catalysts. ZSM-5 (SiO_2_/Al_2_O_3_ mole ratio: 50) was purchased from Zeolyst International. The initial pH of the synthetic dye solution was adjusted using 0.5 M H_2_SO_4_ and 1 M NaOH (Merck). Quantitative estimation of H_2_O_2_ concentration at the end of the experiment was accomplished using peroxide strips.

### Catalyst Preparation

Fe-ZSM-5 was prepared through a newly developed two-step process employing precipitation and thermal decomposition. This process was established and successfully tested for production of a wide range of heterometallic catalysts and their nano-particles. S1 Fig illustrates the understandable process flow diagram for Fe-ZSM-5 synthesis. In the first step, a mono-metallic complex was prepared by reacting iron chloride with 2,2'-bipyridine in 2-propanol. Then, 3.6 g of FeCl_2_.4H_2_O and 8.5 g of 2,2'-bipyridine were each separately dissolved in 20 mL of 2-propanol and mixed dropwise in a round bottom lab-scale crystallizer. The mixture was stirred for 2–3 h at 36°C. Fine crystals of [Fe(byp)_2_]Cl_2_ were then separated from the solution and washed with ether to remove impurities and yield the target at 78%. The produced monometallic complex showed enhanced features, indicating that it could be used directly in a variety of clean energy applications.

In the second step, the Fe-complex was impregnated with ZSM-5 zeolite particles. The concentration of the Fe-complex was adjusted to obtain a 3% Fe (wt/wt) catalyst. After impregnation, the crystalline particles were dried at 100°C for 12 h and then calcined in air at 700°C for 7 h. The temperature was ramped slowly to completely remove the ligand.

Characterization of both the Fe-complex and Fe-ZSM-5 was performed using energy-dispersive X-ray spectroscopy (EDX), scanning electron microscopy (SEM), and fourier transform infrared spectroscopy (FTIR). Surface morphology and composition were additionally analyzed using the Phenom ProX SEM. The surface area method was used to calculate the percentage composition of ZSM-5 and Fe-ZSM-5; the corresponding data is provided in Table 1. Thermal decomposition of the complex caused the formation of nano-sized iron particles on the ZSM-5 matrix.

**Table 1:** EDX analysis of ZSM-5 and Fe-ZSM-5.

Catalyst Clusters	Weight Composition (%)				
	Si	Al	O	C	Fe
Fe-ZSM-5	31.9	1.7	63.3	0.0	3.1
ZSM-5	41.4	2.2	54.3	0.0	-

FTIR studies were conducted using a Perkin Elmer FTIR Spotlight 400. The FTIR spectra for [Fe(bpy)_2_]Cl_2_, ZSM-5, and Fe-ZSM-5 were analyzed for shifts due to coordinate bridging in [Fe(bpy)_2_]Cl_2_ and for the formation of the new complex.

The surface area, pore volume, and pore width of the ZSM support and the Fe-ZSM-5 catalyst are presented in Table 2. The Brunauer-Emmett-Teller (BET) method was employed to calculate specific surface areas and average pore width. N_2_ adsorption-desorption isotherms were used to calculate the total pore volume. Finally, the t-plot method was employed to calculate the micropore volume.

**Table 2:** Pore structure characteristics of the ZSM-5 support and Fe-ZSM-5 catalyst.

Sample	*S*_BET_ (m^2^/g)	*S*_micro_ (m^2^/g)	*S*_ext_ (m^2^/g)	*V*_*t*_ (cm^3^/g)	*V*_micro_ (cm^3^/g)	Pore width (nm)
ZSM-5	293.59	241.75	51.85	0.17	0.11	2.40
Fe-ZSM-5	243.93	112.35	131.57	0.16	0.06	2.68

S_BET_ is the specific surface area, S_ext_ is the external surface area, V_t_ is the total pore volume, and V_micro_ is the micropore volume.

### Experimental

#### Experimental Design

In this work, Minitab 16 was used to program a Taguchi orthogonal array in order to design the oxidation experiments. [Dye]_ini_, Dye/catalyst (wt/wt), catalyst/H_2_O_2_ (wt/wt), pH, reaction time, and temperature were chosen as the control factors while TOC removal, decolorization, and degradation were selected as the responses. This method recommended a total of 27 experiments, a significant improvement over the 729 that would have been required to fully test each factor with relation to the others [36].

Preliminary experiments to determine concentration ranges for the input variables followed the design used in our previous study. For this purpose, 100 mL of dye solution (100 mg/L) was treated with different amounts of the iron catalyst and H_2_O_2_. The amount of catalyst and H_2_O_2_ ranged from 100 to 200 mg and 2 to 5 mL, respectively. The reaction temperature was kept constant at 30°C and the pH was maintained at 3. The initial concentration of the dye and operating range determined for the previously stated variables are provided in Table 3. The L_27_ orthogonal array for the experimental design is given in Table 4 and Table 5.

**Table 3:** Taguchi Design Factors and their levels.

Operating Parameters	Level 1	Level 2	Level 3
Dye (mg/L)	100	150	200
Dye: Catalyst (wt/wt)	0.75	1.00	1.50
H_2_O_2_: Catalyst (wt/wt)	1	1.7	2.5
pH	3	5	9
Temperature (°C)	30	40	50
Time (h)	1	2.5	4

**Table 4:** L_27_ orthogonal design, experimental results, and Taguchi analysis for run 1 to 16,

Run	Dye	Dye/Catalyst	H_2_O_2_/Catalyst	pH	Time	Temperature	Decolorization	Dye Removal	TOC
	(mg/L)	wt/wt	wt/wt		H	(°C)	%	%	%
1	1	3	1	1	1	1	91.35	92.76	46.80
2	1	3	1	1	2	2	97.16	97.73	51.32
3	1	3	1	1	3	3	99.63	99.83	58.30
4	1	2	2	2	1	1	91.63	93.01	37.28
5	1	2	2	2	2	2	99.26	99.52	77.40
6	1	2	2	2	3	3	99.75	99.94	63.96
7	1	1	3	3	1	1	79.24	82.43	51.50
8	1	1	3	3	2	2	95.37	96.19	52.14
9	1	1	3	3	3	3	97.53	98.04	60.24
10	2	3	2	3	1	2	48.84	49.23	26.90
11	2	3	2	3	2	3	72.20	72.32	18.19
12	2	3	2	3	3	1	65.55	65.65	22.19
13	2	2	3	1	1	2	99.07	99.17	48.59
14	2	2	3	1	2	3	99.84	99.94	60.49
15	2	2	3	1	3	1	99.91	99.99	70.50
16	2	1	1	2	1	2	66.15	66.24	22.21

**Table 5:** L27 orthogonal design, experimental results, and Taguchi analysis for run 17 to 27.

Run	Dye	Dye/Catalyst	H_2_O_2_/Catalyst	pH	Time	Temperature	Decolorization	Dye Removal	TOC
	(mg/L)	wt/wt	wt/wt		H	(°C)	%	%	%
17	2	1	1	2	2	3	97.70	97.80	46.10
18	2	1	1	2	3	1	75.47	76.03	27.90
19	3	3	3	2	1	3	58.32	61.71	24.67
20	3	3	3	2	2	1	89.34	90.27	26.37
21	3	3	3	2	3	2	99.07	99.22	54.73
22	3	2	1	3	1	3	65.71	68.51	29.13
23	3	2	1	3	2	1	70.61	73.02	22.29
24	3	2	1	3	3	2	85.78	86.99	18.50
25	3	1	2	1	1	3	98.71	98.89	54.85
26	3	1	2	1	2	1	99.56	99.67	54.38
27	3	1	2	1	3	2	99.79	99.88	67.81

#### Experimentation

Experiments were performed in 500 mL Erlenmeyer flasks equipped with magnetic stirrers. In each experiment, 100 mL of the dye solution was introduced to the flask and adjusted for pH accordingly. The desired amount of catalyst and H_2_O_2_ solution was then added and the resulting mixture was stirred at 250 rpm. The start time was marked as the point when H_2_O_2_ was added. At the end of the experiment, samples were collected from the treated solution, filtered with 0.20 μm Millipore syringe filters, and immediately analyzed.

#### Analysis

A digital pH meter (Cyberscan pH 300, Eutectic Instruments) was used to measure pH values. Quantitative measurement of residual H_2_O_2_ in the treated wastewater was carried out using peroxide test strips (Merck).

All of the raw and treated samples were analyzed using a UV spectrophotometer (Spectroquant Pharo 300, Merck), after which the decolorization and degradation efficiency of the samples was calculated.

$$Decolorization(\%)=\left(1-\frac{Abs_{t}}{Abs_{i}}\right)\times 100$$ (2a)

$$Degradation(\%)=\left(1-\frac{C_{t}}{C_{o}}\right)\times 100$$ (2b)

Where Abs_i_, and Abs_t_, are the UV absorption determined at λ_max_ for each dye and mixture at initial and at a certain time t. Similarly Co and C_t_ are the dye concentrations, calculated at initial and at a certain time t.

In addition to the decolorization measurements, TOC values of the treated samples were analyzed using a TOC Analyzer (SHIMADZU-00077). TOC was calculated as the difference between the total carbon content and inorganic carbon content in the treated sample. TOC removal was calculated as follows:

$$TOC\left(\%\right)=\left(\frac{TOC_{f}}{TOC_{o}}\right)\times 100$$ (3)

Treated samples at optimized conditions were also examined by high performance liquid chromatography (HPLC) using an Agilent technology 1200 series. This analysis has given further details about the degradation products. C18 column (4.6mm × 150 mm × 5μm) at 25°C was used as the separation column for finally produced compounds, with the mobile phase acetonitrile/water (v/v) operated at 60/40 ratio and a flow rate of 1 ml/min. To identify compounds generated from the Fenton degradation of Acid Blue 113, the HPLC system was calibrated using standard analytical reagents namely benzene, phenol, aniline, hydroquinone, benzoquinone, catechol, formic acid, malic acid and oxalic acid. The Fenton-oxidation compounds were identified by comparing their retention times with those of calibrated runs under similar conditions.

#### Taguchi Optimization Steps

The Taguchi method is a robust statistical technique that employs orthogonal arrays for designing experiments. Use of orthogonal arrays allows for an evaluation of factors using the minimum number of experiments, thereby reducing cost and time. The main feature of this method is that it relies on the signal-to-noise (S/N) ratio instead of the precise experimental results. Here, ‘signal’ implies the mean value while ‘Noise’ shows the standard deviation term. Lower variability is ensured by maximizing this ratio. The Taguchi method classifies S/N ratios as smaller is better, normal is better, and larger is better. The formulas representing these ratios are provided in Table 6.

**Table 6:** Theoretical framework of the Taguchi method coupled with principle component analysis (PCA).

**Taguchi Method**		
Step	Detail	Equation
***Step*: *Computing S/N ratios***	Smaller is better, Larger is better	$\frac{S}{N}=-10\;\text{log}\left(\frac{1}{n}\sum_{k=1}^{n}y_{i}^{2}\right)$
	Larger is better	$\frac{S}{N}=-10\;\text{log}\left(\frac{1}{n}\sum_{k=1}^{n}\frac{1}{y_{i}^{2}}\right)$
	Normal is better	$\frac{S}{N}=-10\;\text{log}\left(\frac{1}{n}\sum_{k=1}^{n}\frac{\mu^{2}}{\sigma^{2}}\right)$
***Application of Principal component Analysis to execute output of Taguchi method***		
**Principal Component Analysis**		
***Step 1*: *Normalizing S/N ratios***	• Normalization of S/N ratios (output of Taguchi method) prior to PCA transformation	${\bar{x}}_{i}(n)=\frac{x_{i}(k)-\mu_{i}}{\sigma_{i}}$
***Step 2*: *Finding covariance matrix (C***_***x***_ ***)***	• Computation of covariance matrix to de-correlate S/N ratio Obtained by multiplying the transpose of matrix $\bar{\text{X}}$ with the original matrix	$C_{x}=\frac{1}{n-1}\left(\begin{array}{cccc} {\bar{x}}_{1}{\bar{x}}_{1}^{T} & {\bar{x}}_{1}{\bar{x}}_{2}^{T} & \ldots & {\bar{x}}_{1}{\bar{x}}_{m}^{T}\\ {\bar{x}}_{2}{\bar{x}}_{1}^{T} & {\bar{x}}_{2}{\bar{x}}_{2}^{T} & \ldots & {\bar{x}}_{2}{\bar{x}}_{m}^{T}\\ \vdots & \vdots & \ddots & \vdots \\ {\bar{x}}_{n}{\bar{x}}_{1}^{T} & {\bar{x}}_{n}{\bar{x}}_{2}^{T} & \ldots & {\bar{x}}_{n}{\bar{x}}_{m}^{T} \end{array}\right)$
***Step 3*: *Eigenvalue***	Eigenvalue (λ_i_) corresponding to each response can be computed by solving the determinant of C_x_ In PCA, these are used to find the Eigenvector	det (*C*_*x*_ − *λ*_*i*_ *I*) = 0
***Step 4*: *Eigenvector***	Eigenvector (A_i_) provides information about the data pattern	*C*_*x*_ *A*_*i*_ = *λ*_*i*_ *A*_*i*_
***Step 5*: *Principal component***	Principal Component (Y_i_) is computed to decrease the variance in the reported data.	Y_i_ = X*A_i_,
***Total Principal index (TPCI)***	• TPCI is computed to find out the average factor effect at each level corresponding to each experimental run	[*TPCI*] = *P*_*i*_ *Y*_*i*_ *

* Where P_i_ represents the proportion explained with the principal component

The application of this method is limited to single response processes, and situation gets complicated with multi-response processes like the one analyzed in this study, potentially increasing time and cost. However, it can be combined with other statistical techniques to overcome this limitation; in this case, PCA is especially applicable [35,36].

PCA is a multivariate statistical technique that uses the S/N ratio obtained from the Taguchi analysis. It reduces the dimensionality of a data set consisting of a number of interrelated variables while retaining variation as much as possible in the data sheet [37], and is effected by converting data into components whose contribution to the variability of the original data is easy to identify, so-called principal components. Table 6 outlines the integration of these two methods [38,39].

#### Leaching and Deactivation of Fe-ZSM-5

Fe-ZSM-5 stability was determined by EDX, SEM, and FTIR. Ten different samples of the catalyst were separated from the treated dye solutions through filtration, washed with aqueous methanol, and dried at temperatures up to 200°C. EDX, SEM, BET, and FTIR were then used to examine changes in composition, chemical structure, and morphology caused by metal leaching and in-situ transformation. Catalyst reusability was also assessed.

## Results and Discussion

### Characterization of Fe-ZSM-5

The surface area method was used to calculate the percentage composition of ZSM-5 and Fe-ZSM-5. A significant amount of carbon, nitrogen, and chlorine was present in the monometallic Fe-complex. However, after impregnation with ZSM-5, the coordinated ligand atoms were eliminated through thermal decomposition and calcination (Table 1). In addition, elemental mapping clearly illustrated a uniform distribution of silicon, aluminum, iron, and oxygen atoms inside the catalyst cluster. Silicon and oxygen were the most prominent, while aluminum was the least; however, their distribution was nearly homogenous.

**Fig 2:**
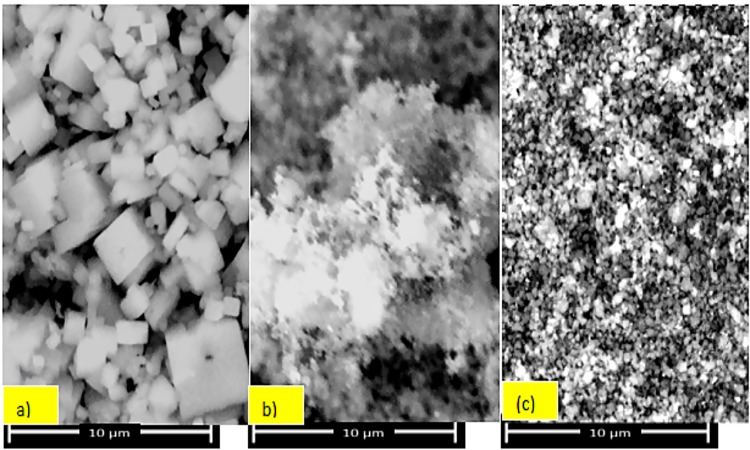
SEM images of (a) the Fe(byp) complex, (b) ZSM-5, and (c) Fe-ZSM-5.

Fig 2 provides SEM data. The crystal morphology of the Fe-complex was significantly changed after calcination. Thermal decomposition of the complex caused the formation of nano-sized iron particles in the ZSM-5 matrix.

**Fig 3:**
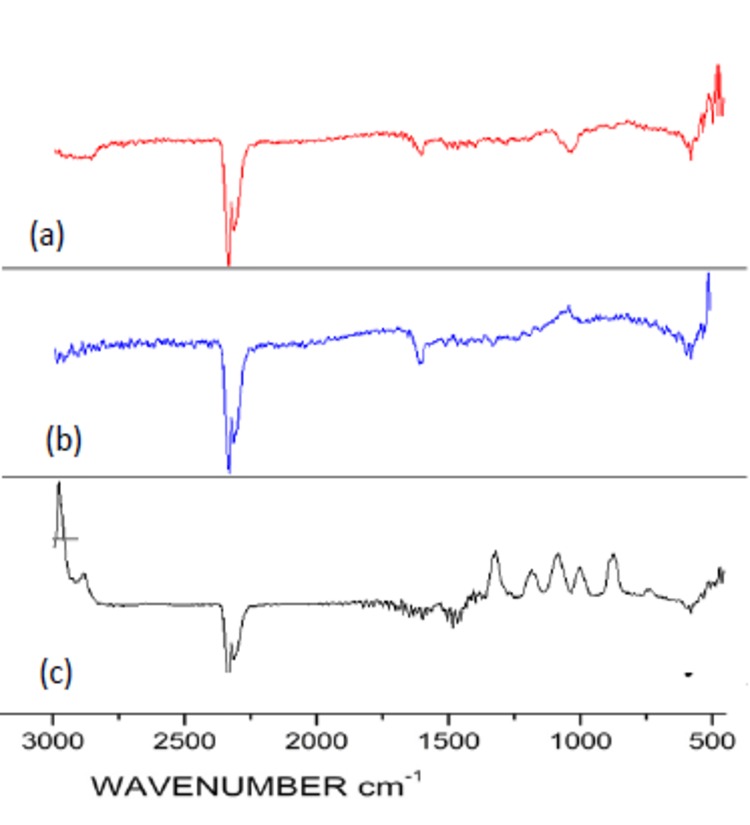
FTIR data for (a) the ZSM-5, (b) Fe-ZSM-5, and (c) Fe(byp) complex.

The FTIR spectra of the [Fe(bpy)_2_]Cl_2_ complex (Fig 3) was studied using 2,2´-bipyridine as a reference in order to track shifts due to coordinate bridging [40]. The major ring stretching vibrations ν(C = C) and ν(C = N) of the free ligand were detected at 1553 and 1579 cm^-1^, respectively. In the Fe-complex, slight shifts lead to corresponding vibrations in the range of 1561–1563 and 1592–1595 cm^-1^, respectively, while the breathing frequency shifted from 991 to 1012 cm^-1^. Similarly, new bands appeared in the range of 1094–1136 and 2886–2888 cm^-1^ for Fe-ZSM-5.

The surface area, pore volume, and pore width of the ZSM support and Fe-ZSM-5 catalyst are presented in Table 2. The BET-surface area of the synthesized Fe-ZSM-5 decreased from 293.59 to 243.93 m^2^/g, while total pore volume decreased from 0.17 to 0.11 cm^3^/g. This change is due to the presence of iron oxide nanoparticles, which filled the ZSM-5 pores and effectively reduced the specific area and pore volume [28]. The average particle size of the loaded material was 12.2987 nm.

### Process Optimization by Coupling the Taguchi Method with PCA

The dye degradation, decolorization, and TOC removal efficiency data, as determined by the combined Taguchi and PCA method employed in this study, are provided in Table 4 and Table 5. A detailed analysis of the tabulated results shows that Fe-ZSM-5 can achieve 99% degradation and 77% mineralization for the selected 100 mg/L dye solution using 1 and 1.7 wt/wt ratios of catalyst and H_2_O_2_ with respect to the dye, respectively. This result is similar to that obtained in our previous work [41], and suggests that iron-based heterogeneous Fenton oxidation could be as efficient as the homogeneous equivalent. Furthermore, at higher dye concentrations of 200 mg/L, the selected protocol gave 68% mineralization and nearly 100% decolorization and dye degradation, respectively.

The responses obtained as a result of Fenton oxidation were then converted to S/N ratios. High degradation efficiency is desirable in Fenton oxidation; therefore, the larger the better S/N ratio was chosen (Table 7 and Table 8). These values were maximized for runs 5, 14, and 15. Because obtaining optimized values for each individual response was not viable, the S/N ratio at each level was optimized and converted to single component called “Total Principal Component Index” by using Principal Component Analysis (PCA).

**Table 7:** The obtained S/N ratios, normalized S/N ratios, and TPCI values for run 1 to 15.

Run	S/N ratio			Normalized S/N ratio			TPCI
	Decolorization	Dye Removal	TOC	Decolorization	Dye Removal	TOC	
1	39.21	39.35	33.40	0.35	0.38	0.33	0.55
2	39.75	39.80	34.21	0.65	0.65	0.53	0.95
3	39.97	39.99	35.31	0.78	0.76	0.81	1.22
4	39.24	39.37	31.43	0.36	0.40	-0.18	0.27
5	39.94	39.96	37.77	0.76	0.75	1.43	1.56
6	39.98	39.99	36.12	0.78	0.77	1.01	1.34
7	37.98	38.32	34.24	-0.36	-0.22	0.54	0.02
8	39.59	39.66	34.34	0.56	0.57	0.56	0.88
9	39.78	39.83	35.60	0.67	0.67	0.88	1.16
10	33.78	33.84	28.60	-2.76	-2.88	-0.89	-3.30
11	37.17	37.19	25.19	-0.82	-0.90	-1.76	-1.85
12	36.33	36.34	26.92	-1.30	-1.40	-1.32	-2.09
13	39.92	39.93	33.73	0.75	0.73	0.41	0.96
14	39.99	39.99	37.03	0.79	0.77	1.24	1.48
15	39.99	40.00	35.56	0.79	0.77	0.87	1.27

**Table 8:** The obtained S/N ratios, normalized S/N ratios, and TPCI values for run 16 to 27.

Run	S/N ratio			Normalized S/N ratio			TPCI
	Decolorization	Dye Removal	TOC	Decolorization	Dye Removal	TOC	
16	36.41	36.42	26.93	-1.26	-1.35	-1.32	-2.04
17	39.80	39.81	33.27	0.68	0.66	0.29	0.83
18	37.55	37.62	28.91	-0.60	-0.64	-0.81	-1.08
19	35.32	35.81	27.84	-1.88	-1.71	-1.09	-2.40
20	39.02	39.11	28.42	0.24	0.24	-0.94	-0.30
21	39.92	39.93	34.76	0.75	0.73	0.67	1.11
22	36.35	36.72	29.29	-1.29	-1.18	-0.72	-1.63
23	36.98	37.27	26.96	-0.93	-0.85	-1.31	-1.63
24	38.67	38.79	25.34	0.03	0.05	-1.72	-0.94
25	39.89	39.90	34.78	0.73	0.71	0.67	1.10
26	39.96	39.97	34.71	0.77	0.75	0.66	1.13
27	39.98	39.99	36.63	0.78	0.76	1.14	1.42

First, the S/N ratio at each level was normalized. Subsequently, PCA was applied to the normalized data, using matrix $\bar{\text{X}}$. After the stepwise execution, matrix $\bar{\text{X}}$ was converted into covariance matrix C_x_, which was used to compute Eigenvalues for these responses.

Eigenvalues of the three principal components and the corresponding Eigenvectors are given in Table 9. The Eigenvalues obtained by PCA were 2.69, 0.31, and 0.002. This procedure transformed the normalized S/N ratios into a set of uncorrelated principal components; the value with an Eigenvalue greater than 1 was then chosen [42].

**Table 9:** Eigenvalues and Eigenvectors obtained through PCA processing of the normalized S/N ratios.

Principal Component	Eigenvalue	Proportion (%)	Cumulative (%)	Eigenvector
First	2.69	89	89	[0.595, 0.596, 0.539]
Second	0.31	10	99.9	[0.383, -0.38, 0.842]
Third	0.002	1	100	[-0.706, 0.708, -0.0002]

The Eigenvalue of the first principal component can be used to explain the performance characteristics of the Fenton oxidation. In this case, the first two Eigenvalues were used to compute the TPCI, since together they accounted for 99% of the observed variance. Larger TPCI values imply better process performance; based on the obtained values, it was therefore observed that pH significantly contributed to efficiency. Overall, the following trend was observed for influence of the assessed parameters:

pH>Dye>Time>DyeFe+2>H2O2Fe+2>Temperature (4)

**Fig 4:**
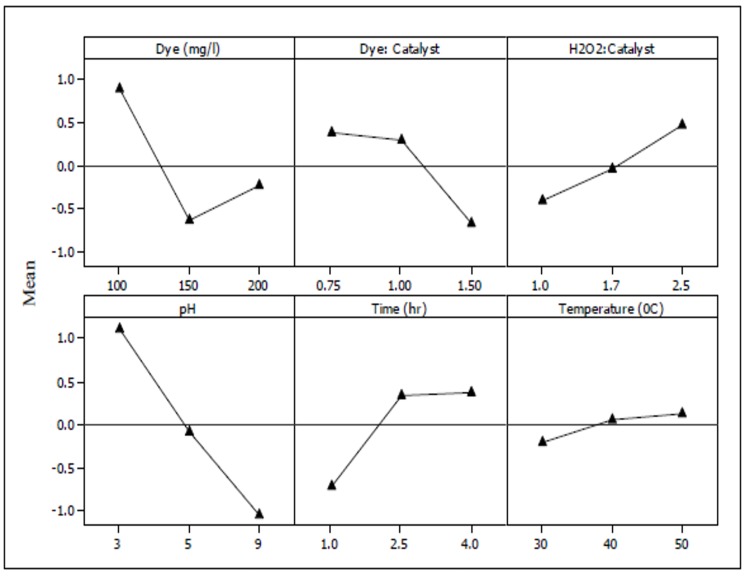
Mean TPCI plots with respect to parameter changes.

The TPCI values listed in Table 10 and the corresponding data in Fig 4 could be used to optimize the operating parameters. In this vein, those parameters that provided the maximum TPCI values were selected (Table 10). The optimized values showed that consumption of iron catalyst was reduced by 90% in comparison with our previous work [41]; however, energy requirements increased. Since TPCI analysis ranked temperature at the lowest number in the list, therefore 30°C was selected as the optimized value instead of 50°C.

**Table 10:** List of TPCI responses.

Level	Dye	Dye: Fe	H_2_O_2_:Fe^+2^	pH	Time (h)	Temperature (°C)
1	0.88401	0.37928	-0.41912	1.11866	-0.71866	-0.20613
2	-0.64688	0.29935	-0.04612	-0.07721	0.33794	0.06688
3	-0.23708	-0.67857	0.46530	-1.04139	0.38078	0.13931
Delta	1.53089	1.05784	0.88442	2.16005	1.09944	0.34545
Rank	2	4	5	1	3	6

Delta: difference of max-min values for each column; Rank: Order of factor significance.

Reaction efficiency was then assessed at the optimized conditions. These experiments were also repeated at higher dye concentrations; the results are summarized in Table 11. At higher dye concentrations, decrease in TOC removal was observed. This is because at higher concentration, degradation rate is low due to non-availability of hydroxyl radicals [43]. Overall, it was confirmed that the heterogeneous reaction performs just as well as the homogeneous one, with the added advantage that it reduces consumption of the iron catalyst [44]. To identify different compounds generated at optimized conditions, the HPLC system was employed. Traces of carboxylic acid, benzoquinone and aniline were detected corresponding to the peaks at ca. 1.92 min, 2.420 min and 5.97 min, respectively. Aniline formation would be in agreement with the oxidative hemolytic splitting of dye amino benzene moiety making a °NH-C_6_H_5_ radical which could then abstract an amino hydrogen atom from other dye molecule [45].

$$C-NH-C_{6}H_{5}+N^{o}H-C_{6}H_{5}\;\to \;C-N^{o}-C_{6}H_{5}+\;NH_{2}-C_{6}H_{5}$$ (5)

**Table 11:** Optimized values for heterogeneous Fenton oxidation.

Dye	Dye/Catalyst	H_2_O_2_/Catalyst	pH	Time	Temperature	Decolorization	Dye Removal	TOC
(mg/L)	wt/wt	wt/wt		h	(°C)	%	%	%
100	0.75	2.5	3	4	30	99.7	99.8	77
200	0.75	2.5	3	4	30	99.6	99.7	69
250	0.75	2.5	3	4	30	99.2	99.3	70
500	0.75	2.5	3	4	30	99.4	99.5	71
1,000	0.75	2.5	3	4	30	99.1	99.1	71.5

The other identified compound was benzoquinone which is often found in the degradation of benzene and phenol [45,46]. Further oxidation and splitting of benzoquinone ring resulted into formation of carboxylic acids, particularly maleic acid and fumaric acid [46]. These results highlight the utility of this analytical method, which clearly can be used for the systematic investigation and optimization of multiresponse systems.

### Analysis of Variance (ANOVA)

ANOVA is used in the Taguchi method to evaluate the experimental results and to determine the contribution of each factor to their overall variance [41]. ANOVA is similar to regression analysis, which is employed to determine the relationship between response variables and one of more responses. These results are provided in Table 12.

**Table 12:** ANOVA analysis.

Factor	Degree of Freedom	Sum of Squares	Mean Squares	F-ratio	p-value	Percent Contribution (%)
Dye	2	11.31	5.65	3.02	0.068	20.08
Dye/Catalyst	2	6.25	3.12	1.50	0.244	11.09
H_2_O_2_/ Catalyst	2	3.55	1.77	0.81	0.458	6.30
pH	2	21.08	10.54	7.18	0.004	37.44
Time	2	6.98	3.49	1.70	0.204	12.40
Temperature	2	0.60	0.30	0.13	0.880	1.06

Again, pH was the most significant variable, with a contribution of 37.44%; meanwhile, temperature was the least significant, with a contribution of 1.06%. The ANOVA results are in agreement with the TPCI results listed in Table 10. Based on the TPCI values, the regression analysis equation was calculated as follows:

$$TPCI\;=2.61-0.0112\;Dye-1.49\;Dye\;/Catalyst\;+0.591\;H_{2}O_{2}\;/Catalyst\;-0.343\;pH+0.366\;t+0.01731\;T$$ (6)

### Interaction of Operating Parameters

Fenton oxidation efficiency strongly depends not only on the process parameters themselves, but on the interaction between them. This section addresses the effect of that interaction and their effects on TOC removal and dye degradation.

#### Interaction Between Operating Parameters with Respect to TOC Removal

**Fig 5:**
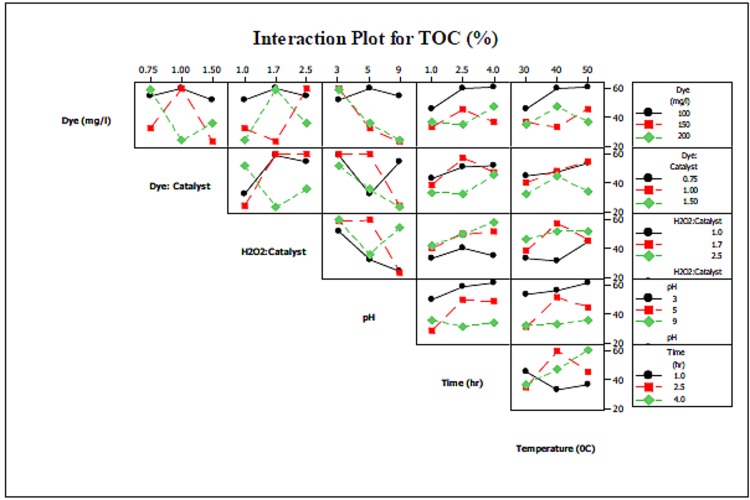
Interaction of parameters with respect to TOC.

An interaction plot between the parameters Dye and Dye/Catalyst shows that an increase in the Dye/Catalyst ratio reduced mineralization efficiency for a constant dye concentration (Fig 5). Higher values of both of these parameters resulted in a significant decrease in mineralization efficiency. Maximum mineralization efficiency was achieved at a dye concentration of 150 mg/L and a Dye/Catalyst ratio of 1.00. On the other hand, at constant dye concentrations, an increase in the H_2_O_2_/Catalyst ratio reduced TOC removal. Similar trends were observed between the Dye/Catalyst and H_2_O_2_/Catalyst ratios.

These results are consistent with the observation that at higher values for the Dye, Dye/Catalyst, and H_2_O_2_/Catalyst parameters, the amount of catalyst added is greatly reduced compared to added H_2_O_2_. This means that less amounts of the catalyst are available to interact with the H_2_O_2_, such that residual H_2_O_2_ will react with the oxidized form of Fe^+3^ or scavenge hydroxyl radicals through the following reactions [47]:

$$Fe^{+3}+H_{2}O_{2}\to Fe^{+3}+HO\overset{•}{O}+H^{+}$$ (7)

$$H\overset{•}{O}+H_{2}O_{2}\to HO\overset{•}{O}+H_{2}O$$ (8)

This results in a significant decrease in mineralization efficiency. This also makes the effect of pH on mineralization efficiency even more obvious than it already was; for all values studied of the operating parameters, lower pH values were favored, while higher pH values decreased mineralization efficiency. This is because ferrous ions are easily converted into ferric ions, which have a tendency to produce ferric-hydroxo complexes with H_2_O_2_ at higher pH [48].

Another parameter that is commonly addressed is the time required to elicit Fenton oxidation. In this process, reaction time is influenced by the rate of HO radical formation, which is in turn produced by the interaction between iron salt and H_2_O_2_. As observed from the graph in Fig 5, at lower values for Dye and Dye/Catalyst, mineralization efficiency slowly increased with time as more H_2_O_2_ was made available. In addition, excess amounts of H_2_O_2_ may react with ferric salts after complete consumption of Fe^+2^; however, this reaction is slow, which is why at the end of reaction, mineralization efficiency decreased. However, at higher operating parameters, with the exception of the H_2_O_2_/Catalyst ratio, mineralization efficiency decreased with time. This was attributed to the fact that with excess amounts of H_2_O_2_, residual H_2_O_2_ reacts with ferric iron present on the surface of the heterogeneous catalyst and degrades the intermediate products formed as a result of the initial degradation reaction [49]. On the other hand, mineralization efficiency increased with temperature, with an approximate 20% increase observed when temperature rose from 30 to 50°C. However, again, this increase was minimal at higher temperatures, since such a change increases the reaction rate between H_2_O_2_ and the iron catalyst and thus decreases the concentration of hydroxyl radicals [50]. Finally, in several experimental runs, such as run 6 and 11, reduction in TOC removal efficiency was observed with an increase in temperature. This may be due to decomposition of H_2_O_2_ at high temperatures.

#### Interaction of Factors with Respect to Dye Decolorization Efficiency

Fenton oxidation induces rapid decolorization [20]. This is because the color content of the dye is linked to the chromophore, or the conjugated unsaturated double bond present in the dye structure. The azo bonds in particular are active and can be easily broken by highly oxidative hydroxyl radical [51,52]. This explains why decolorization efficiency is generally higher than mineralization efficiency. As seen in Table 4, over 99% dye degradation was achieved in runs 14 and 15.

**Fig 6:**
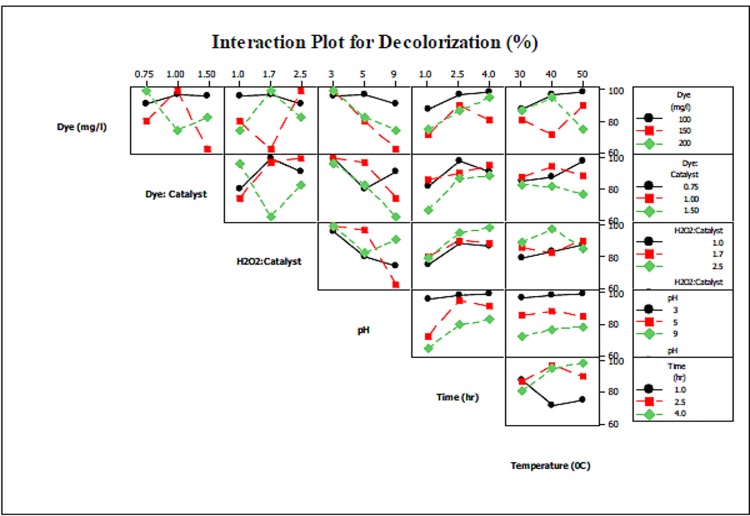
Interaction of parameters with respect to decolorization.

**Fig 7:**
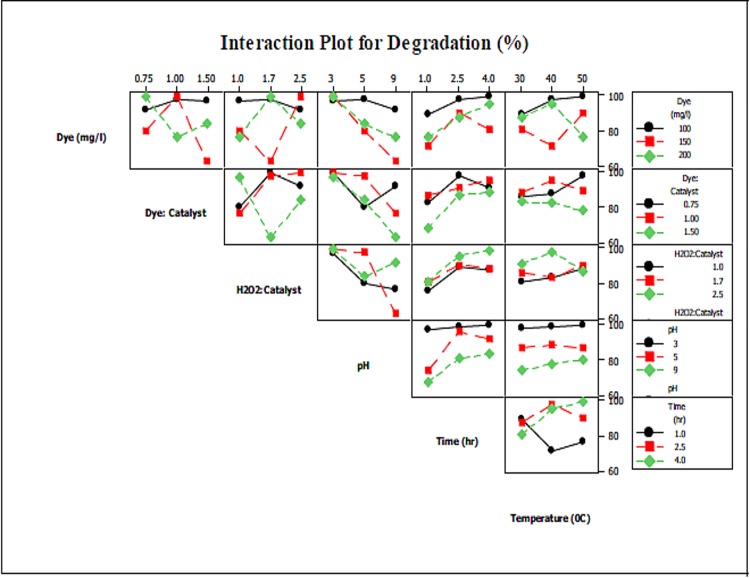
Interaction of parameters with respect to degradation.

Nevertheless, the degree of decolorization varies with changes in the operating parameters. Interaction plots for dye degradation and decolorization efficiencies are shown in Figs 6 and 7, respectively. Similar trends to those observed for TOC removal efficiency are present here; as dye degradation and decolorization efficiencies are directly linked with each other, this section treats the observed phenomenon for only dye degradation.

Overall, dye degradation efficiency was higher at lower dye concentrations and Dye/Catalyst ratios. This is due to the fact that at higher dye concentrations and lower Dye/Catalyst ratios, the amount of catalyst added is higher, which may result in rapid production of hydroxyl radicals and adsorption of dye during the neutralization stage. On the other hand, an increase in dye concentration and the H_2_O_2_/Catalyst ratio reduced decolorization efficiency. This observation can be supported by the fact that at higher H_2_O_2_/Catalyst ratios, excess amounts of H_2_O_2_ are added in comparison with the amount of catalyst. This results in reduced production of hydroxyl radicals, which eventually leads to the observed effect.

As observed in Fig 6, pH had an inverse relationship with dye decolorization efficiency. This is because at higher pH values, H_2_O_2_ decomposes to O_2_ and ferrous ions are converted into ferric-hydroxo complexes. Previous research shows that the decolorization process is rapid in nature and that complete decolorization can be achieved within 10 min. It is also evident from the Fig 6 that increase in time has no significant effect on dye decolorization efficiency. Although temperature was still found to be the least significant, its effect on dye decolorization efficiency was obvious in most cases. For instance, at higher dye concentration and temperature, dye degradation increased with time, though this effect may be due to thermal decomposition of the dye. On the other hand, lower dye concentrations and higher levels of the H_2_O_2_/Catalyst and Dye/Catalyst ratios resulted in decreased dye degradation efficiency. This is because higher temperatures thermally decompose H_2_O_2_, thus reducing the availability of H_2_O_2_ for hydroxyl radical production.

#### Long Term Stability and Reusability of Fe-ZSM-5

In addition to the heterogeneous catalytic activity of the synthesized catalyst, it is important to consider stability and reusability as well. The decreased leaching of iron ions from zeolites during wastewater treatment is an indication of catalyst stability [28], so this parameter was tracked by EDX, SEM, and FTIR.

**Fig 8:**
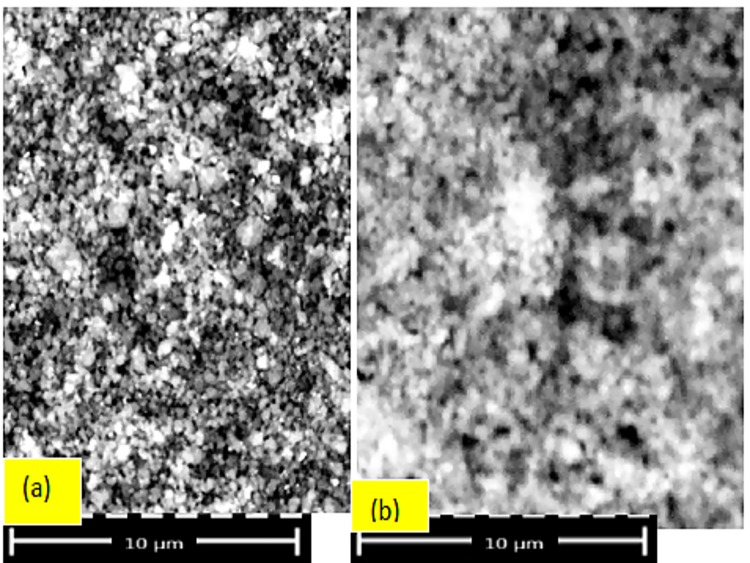
SEM images of the (a) fresh and (b) spent Fe-ZSM-5.

Ten different samples of the spent Fe-ZSM-5 were collected from the treated wastewater at the same temperature at which the reaction was performed. The samples were then washed with methanol and dried at 200°C before being analyzed by EDX. This method allows indirect measurement of the presence of iron ions leached into solution. In the majority of cases, less than 2% of leaching was observed; however, at a pH of 3 and at 30°C, this value increased to 2–3%, further jumping to 6–7% once temperature was increased to 50°C. This shows that catalysis mainly on the surface of the particles, instead of on the leached iron although a small amount of iron was present in the aqueous phase. Furthermore, SEM data (Fig 8) shows no significant difference in the crystal morphology between the fresh and spent catalysts.

**Fig 9:**
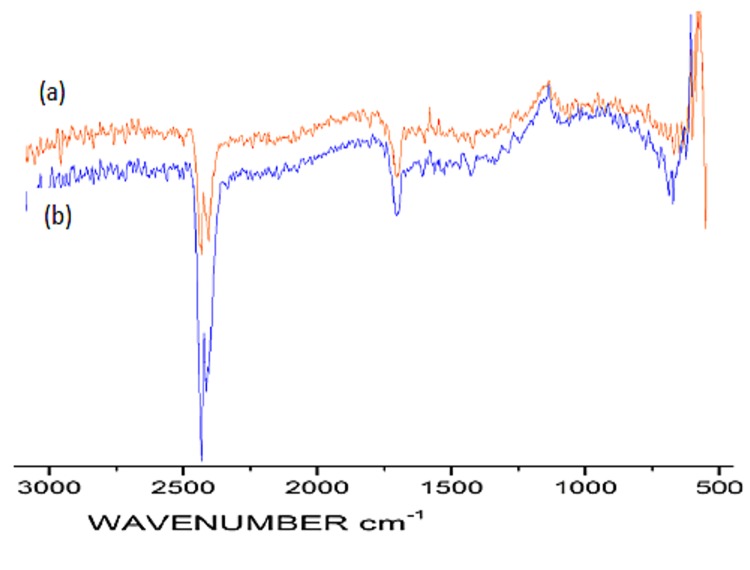
FTIR spectra of the (a) fresh and (b) spent Fe-ZSM-5.

In addition, the FTIR spectra of the fresh and spent catalysts were also observed (Fig 9), yet showed no significant change in the chemical structure.

To test the reusability, the recovered catalyst was subjected to the same process conditions after being dried at 200°C for 3 h. Three trials were carried out, all of which indicated continued stability. This indicated that the catalytic behavior of the developed catalyst was reproducible in consecutive experiments without a significant drop in its efficiency. This shows that catalyst deactivation, either due to iron leaching or in-situ transformation, is insignificant.

#### Comparison of the Fe-ZSM-5 with Existing Catalysts

The efficiency of the synthesized catalyst was compared with other iron based catalysts used for the degradation of Acid Blue 113. However, from literature, authors could not able to find the data regarding the degradation of Acid Blue 113 with Fe-ZSM-5. Therefore, the comparison includes the efficiencies of the different catalysts used for the degradation of Acid Blue 113 [Table 13] and the efficiencies of Fe-ZSM-5 used against different dyes (Table 14). For the readers ease, the comparisons values of chemicals are rearranged setting a basis of one liter of dye solution.

**Table 13:** Comparison of synthesized Fe-ZSM-5 with other catalysts used for the degradation of Acid Blue 113.

Catalyst	Dye	Fe^+2^	H_2_O_2_	pH	Decolorization	TOC Removal	Reference
	(mg/L)	mg/L	mg/L		%	%	
Fe-ZSM-5	100	39	3,333	3	99.7	77	Present study
Fe-ZSM-5	200	78	6,666	3	99.6	69	Present study
FeSO_4_.7H_2_O	100	116	7,326	2	97.6	50.3	[36]
FeSO_4_.7H_2_O	200	116	7,326	5	84.3	60.8	[36]
NZVI	100	0.1	-	3	99.2	12.6	[12]
NZVI	200	0.5	-	3	85.5	11.5	[12]

Fe-ZSM-5 (Iron Zeolite Socony Mobil-5), and NZVI (Nano Zero-Valent Iron)

**Table 14:** Efficiency comparison chart of different Fe-ZSM-5 catalysts used for the degradation of dyes.

Dye	Dye Conc.	FeZSM-5	H_2_O_2_	pH	Time	Decolorization	TOC/COD Removal	Reference
	(mg/L)	Wt (mg/L)	Wt (mg/L)		H	%	%	
AB113	100	1,330	3,333	3	4	99.7	77 (TOC)	Present study
RG	100	1,000	5,440	4	2.5	99.0	80 (TOC)	[44]
OII	35	333	680	3	3	91.0	36 (TOC)	[53]
RR141	100	2,000	9,078	3.5	2	100	81 (COD)	[55]
RR141	100	1,000	1,122	3.5	2	57	0 (COD)	[55]
OII	50	Not available	9,067	7	2	99.9	55 (COD)	[54]

AB113 (Acid Blue 113), RG(Rodamine G), OII(Orange II), and RR141 (Reactive Red 141)

In case of Acid Blue 113 degradation, with presently synthesized Fe-ZSM-5, lower amount of H_2_O_2_ was required to degrade the dye (Table 13). Consumption of iron salt and H_2_O_2_ were greatly reduced more than 90% and 60%, respectively. The efficiency of Fe-ZSM-5 was also compared with resin supported nano zero-valent iron (NZVI) particles [42]. Although these supported particles were efficient up to some extent in color removal (80–99%) of Acid Blue 113 and their efficiency was found to be mainly depend upon the pH and catalyst loading. However, maximum TOC removal was noted below 12.6%, which is significantly less than the synthesized Fe-ZSM-5 (Table 13).

Similarly, Table 14 gives a comparison of efficiencies related to different Fe-ZSM-5 catalysts. The precise assessment is not possible as dyes were different in all cases. Table 14 reveals that Fe-ZSM-5 catalysts prepared by ion exchanged methods are adequate for the decolorization of dyes [53–55]. Queiros et al. [53] used lesser amount of catalyst as compared to this work. Similarly Bolova et al. [54] and Queiros et al. [53] used lower concentrations of dyes (35 mg/L and 50mg/L) to evaluate the activities of their synthesized catalysts. At lowered initial dye concentrations, degradation rate is usually high due to availability of hydroxyl radicals [43]. Despite of lower concentrations, the reported values of TOC removal were significantly smaller than this work (Table 14).

With excess amount of catalyst and H_2_O_2,_ and relatively high temperature (333K), Yaman and Gunduz [55] got 100% decolorization and 81% of COD removal for Reactive Red 141. However, the degradation efficiency drastically decreased (0% of COD removal) when ambient conditions were adopted. Similar trend (52% of COD removal) was found with lowering catalyst concentration (1.0 g/L).

Prihod’ko et al. [44] used improved Fe-exchanged zeolites for the degradation of Rodamine G dye. Remarkable TOC removal (80%) has been reported with 100% dye decolorization in a relatively shorter reaction time (2.5 h). Amount of H_2_O_2_ used was in the range of 5.44–6.8 g/L (0.16–0.20 mol/L), which is almost twice of this work. In addition to excess H_2_O_2,_ longer reaction time (3–4 days) is required for the synthesis of improved Fe-exchanged zeolites as compared to two-step process [31]. This comparative study reveals that Fe-ZSM-5 synthesized through two-step is efficient and economical.

#### Efficiency of Synthesized Fe-ZSM-5 for Mixture of Dyes

**Fig 10:**
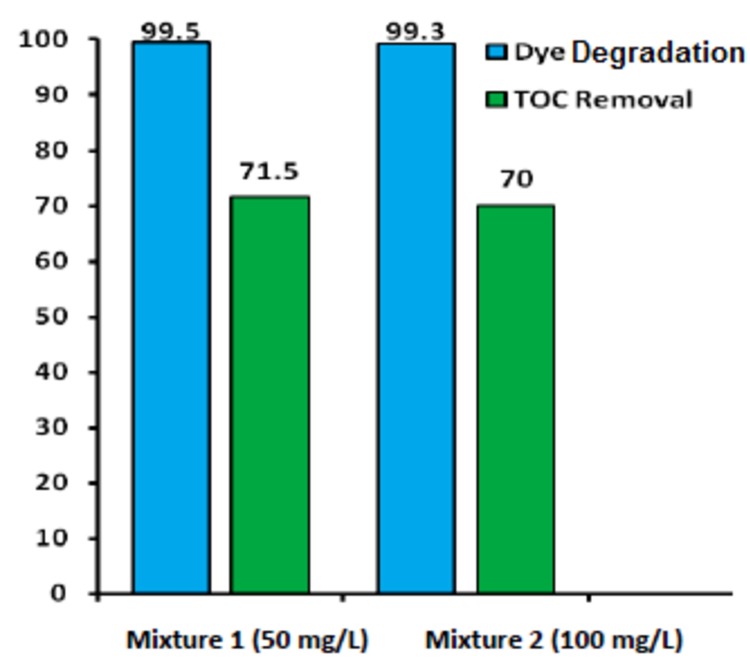
Degradation and TOC removal of mixture of dyes using Fe-ZSM-5.

A set of experiments were performed for the mixtures of three dyes containing 50%, 25% and 25% of Acid Blue 113, Reactive Black 5 and Methyl Orange, respectively. Two stock solutions with net concentrations of 50mg/L and 100mg/L were prepared and treated at optimized values (Table 11). Decolorization and dye removal was combined as dye degradation, and was measured by recording average variations in absorbance at λ_max_ corresponding to each dye. Fig 10 illustrates the clear picture of degradation and TOC removal. The measured values of dye degradation were 99.5% and 99.3%, while TOC removals were 71.5% and 70%. A reduction of 6–7% in TOC removal indicates that the mixture mineralization is relatively complex phenomena. Other reason may be the presence of Methyl Orange, the degradation of which is fevered by alkaline medium particularly at pH 9 [56]. However, overall performance of the synthesized catalyst was remained excellent for the dyes mixtures.

## Conclusions

In this study, Fe-ZSM-5 was synthesized using a newly developed 2-step process, after which the catalyst was characterized by SEM, EDX, FTIR, and BET. The performance of the developed catalyst was analyzed by performing Fenton oxidation on the azo dye Acid Blue 113. The catalyst has enhanced shown activity, yielding over 99% degradation and decolorization and 77% mineralization efficiency under optimized conditions (Dye/Catalyst = 1 and H_2_O_2_/Catalyst = 1.7 (wt/wt)). Furthermore, at higher dye concentrations (200–1,000 mg/L), and in mixture of dyes, the mineralization efficiency was in the range of 69–71%. Applying Fe-ZSM-5 heterogeneous Fenton oxidation is economical, as 90% consumption of catalyst was reduced. Stability testing with respect to metal leaching and reusability showed that less than 2% of the iron was leached. Thus, the present study proved that the developed catalyst is efficient and economical. In the future, the authors aim to investigate the efficiency of this catalyst against other organic pollutants. Moreover, the addition of ZnO and CuO nanoparticles is also under consideration in order to further improve performance.

## Supporting Information

S1 Fig Process flow diagram for the synthesis and application of Fe-ZSM-5. (DOCX)
