# Supplementary figures and images for: Enhancement of Treatment Efficiency of Recalcitrant Wastewater Containing Textile Dyes Using a Newly Developed Iron Zeolite Socony Mobil-5 Heterogeneous Catalyst
Source: PLoS One. 2015 Oct 30;10(10):e0141348. doi: 10.1371/journal.pone.0141348 (PMC4627726; doi:10.1371/journal.pone.0141348)

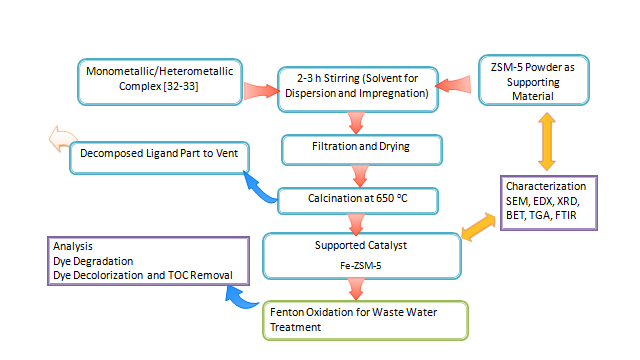

Supplement: S1 Fig — (DOCX) [file pone.0141348.s001.docx]
